# Evolutionary history and association with seaweeds shape the genomes and metabolisms of marine bacteria

**DOI:** 10.1128/msphere.00996-24

**Published:** 2025-06-02

**Authors:** Catherine A. Pfister, Johanna Berlinghof, Maximiliana Bogan, Ulisse Cardini, Angélique Gobet, Pauline Hamon-Giraud, Jessica Hart, Natalia Jimenez, Anne Siegel, Emma Stanfield, Marine Vallet, Catherine Leblanc, Coralie Rousseau, François Thomas, Willem Stock, Simon M. Dittami

**Affiliations:** 1 Department of Ecology & Evolution, The University of Chicago 166953, Chicago, Illinois, USA; 2 Genoa Marine Center, Stazione Zoologica Anton Dohrn, Genoa, Italy; 3 Department of Marine Ecology, University of Bremen 9168, Bremen, Germany; 4 MARBEC, Univ Montpellier, CNRS, Ifremer, IRD, Sète, France; 5 CNRS, Inria, IRISA (UMR 6074), University of Rennes 27079, Rennes, Brittany, France; 6 Institute for Biological and Medical Engineering, Schools of Engineering, Medicine and Biological Sciences, Pontificia Universidad Católica de Chile 28033, Santiago, Chile; 7 Department of Chemical and Bioprocess Engineering, School of Engineering, Pontificia Universidad Católica de Chile 117440, Santiago, Chile; 8 Millenium Institute Marine Agronomy of Seaweed Holobionts (MASH, NCN2021033), Puerto Montt, Chile; 9 Institute for Inorganic and Analytical Chemistry, Friedrich Schiller University Jena 9378, Jena, Thuringia, Germany; 10 Max Planck Fellow Group Plankton Community Interactions, Max Planck Institute for Chemical Ecology 28298, Jena, Thuringia, Germany; 11 Integrative Biology of Marine Models (LBI2M, UMR 8227), CNRS, Station Biologique de Roscoff (SBR), Sorbonne University 129722 , Roscoff, Brittany, France; 12 Phycology Research Group, Department of Biology, Ghent University 26656, Ghent, Flanders, Belgium; Clemson University, Clemson, South Carolina, USA

**Keywords:** host-microbe, nonhuman microbiome, marine bacteria, microbial metabolism, bacterial genomes

## Abstract

Seaweeds harbor a rich diversity of bacteria, providing them with metabolic resources and a surface for attachment and biofilm development. The host’s unique environment potentially shapes the bacterial genomes and promotes adaptations for a symbiotic lifestyle. To investigate whether the genomes of seaweed-associated bacteria are genetically and metabolically distinct from their close free-living relatives in seawater, we compared both the seaweed-associated and free-living counterparts of 72 bacterial genera across 16 seaweed hosts using whole-genome sequences or high-quality metagenome-assembled genomes. While taxonomic affiliation strongly influenced genome characteristics such as GC content, gene number, and size, host association had a lower effect overall. A reduced genome size was suggested only in *Nereocystis luetkeana*-associated microbes, while only *Ascophyllum nodosum*-associated bacteria had an increased GC content. Metabolic adaptations were indicated from the genomes of seaweed-associated bacteria, including enriched pathways for B vitamin production, complex carbohydrate utilization, and amino acid biosynthesis. In particular, *Flavobacteriia* showed the most pronounced differences between host-associated and free-living strains. We further hypothesized that bacteria associated with seaweed might have evolved to complement their host’s metabolism and tested this inference by analyzing the genomes of both the seaweed *Ectocarpus subulatus* and its 28 bacterial associates but found no evidence for such complementarity. Our analyses of 72 paired bacterial genomes highlighted significant metabolic differences in seaweed-associated strains with implications for carbon, nitrogen, and sulfur cycling in the coastal ocean.

**IMPORTANCE:**

We hypothesized that the unique environment of seaweeds in coastal oceans shapes bacterial genomes and promotes a symbiotic lifestyle. We compared the genomes of bacteria isolated from seaweed with bacteria from the same genus found free-living in seawater. For genome features that included the number of genes, the size of the genome, and the GC content, taxonomy was of greater importance than bacterial lifestyle. When we compared metabolic abilities, we again found a strong effect of taxonomy in determining metabolism. Although several metabolic pathways differed between free-living and host-associated bacteria, this was especially prominent for *Flavobacteriia* in the phylum *Bacteroidota*. Notably, bacteria living on seaweeds had an increased occurrence of genes for B vitamin synthesis, complex carbohydrate use, and nitrogen uptake, indicating that bacterial genomes reflect both their evolutionary history and the current environment they inhabit.

## INTRODUCTION

Microbial associations with diverse marine hosts are increasingly recognized, and transitions from a free-living lifestyle to a symbiotic one with hosts have been demonstrated (1, 2). The degree to which the holobiont, i.e., host and its associated microbes, interacts physically, genetically, and metabolically is an area of active investigation, though it is better understood for strong symbiotic relationships, such as the bobtail squid and its *Vibrio* bacterium (3). Indeed, a major evolutionary transition occurs when independent entities combine to form a more complex, functionally integrated organism (4), including the recent discovery of an N_2_-fixing organelle, or “nitroplast,” in a marine microalga (5).

While significant attention has been devoted to endosymbiotic mutualisms, little is known about the genetic and metabolic dependencies between hosts and microbial partners that may not be obligate. Marine bacteria are highly diverse in the ocean and can be found as free-living cells (e.g., references 6, 7) and in cell-to-cell association with eukaryotic hosts (8–10). Marine primary producers, such as phytoplankton or seaweeds, host and interact with complex bacterial consortia (11–15). Some fundamental processes, such as carbon fixation, have been demonstrated to be a property of the holobiont, resulting from a mutualistic interaction between the host and bacteria (16, 17). Coastal seaweeds host a high diversity of bacteria (18), with cell abundances estimated at 10^5^–10^7^ cells per square centimeter of algal tissue (19), and host-associated bacteria engage in many functions to support their seaweed hosts. For instance, these bacteria can determine the host phenotype (20), contribute to host nutrient acquisition (21, 22), control algal morphogenesis (20, 23), and protect against the establishment of pathogenic bacteria (24). Marine bacteria also contribute to seaweed acclimation to salinity changes (25). While bacterial consortia are affected by heat stress (26), they may not provide further support to their host when challenged (27). Seaweed-associated bacteria also degrade algal compounds (10) and induce diseases (28). They might be able to switch from a mutualistic to pathogenic lifestyle through their interactions with algal hosts as observed in alga-phytoplankton interactions (29, 30).

While there are scant examples where marine hosts and their microbiomes are fully described and sequenced, corals and their symbionts are an exception. The association of coral with Symbiodiniaceae dinoflagellates and bacteria is a relevant model of complementarity in a holobiont: the growing coral host is provided with B vitamins and photosynthetic carbon from the alga, while bacteria contribute to nutrient acquisition (31). Seaweeds associate with a community of restricted, primarily beneficial bacteria (32, 33), and these host-bacterial interactions might also have specific metabolic networks in which the metabolic capabilities of the host are complementary to what the associated microbe needs and vice versa. In seaweeds, the brown alga *Ectocarpus subulatus* represents an example where the genome of the host (34) and associated bacteria (35, 36) are known, enabling tests of complementarity by inferring metabolic abilities from host-associated genomes (37) versus their free-living counterparts.

The genome size of bacteria has been shown to be reduced, sometimes to extremes, in the transition from a free-living to an obligate endosymbiotic lifestyle (38, 39). A broad survey of genome changes demonstrates that an association with animal hosts decreases genome size while key genes may be acquired through horizontal gene transfer (40). The Black Queen Hypothesis is based on the premise that there is an advantage conferred by carrying genetic material only for essential functions. Gene functions related to public goods, for example, can instead be leveraged from other species, and their maintenance can be selected against due to their metabolic cost (41). Host association may bring different bacterial taxa in close association and foster cross-feeding and species interactions based on auxotrophy. Consistent with the cost hypothesis, microbes associated with phytoplankton have reduced genome size in nutrient-poor lakes compared to those in nutrient-rich lakes (42). We hypothesized that the potentially high density and spatial proximity concentration of bacteria on seaweed hosts (19) could foster cross-feeding and drive gene loss in host-associated bacteria.

How genomes are constructed may also differ for host-associated microbes. The nucleotides that bacteria use to construct their genomes vary. GC bonds use an additional nitrogen atom and could be more costly (43), perhaps explaining increased GC content in areas with increased nitrogen availability (44). Taxa with low GC content are associated with increased use of low-nitrogen amino acids, while higher GC content is associated with the use of more nitrogen-rich amino acids (45). When bacterial taxa are influenced by “resource-driven selection” at the genome level (46), the GC content might be related to whether bacteria were host-associated. The propensity for an AT mutational bias is striking in beneficial endosymbiosis, where a reduction in genome size is accompanied by a decrease in GC content over evolutionary time and with increasing integration of host and symbiont (38). Carbon sources have also been linked to GC content, with increased growth on sugars (generally rich in carbon and depleted in nitrogen) related to lower GC levels in bacterial genomes (45). If seaweed hosts provide specific amino acids, polysaccharides, or nitrogen-based resources and GC content is related to the energy sources, we hypothesize that bacteria associated with seaweeds might have distinct GC content compared to free-living bacteria found in seawater.

Seaweeds release dissolved organic compounds, including dissolved organic carbon (47, 48), and a diversity of mono- and polysaccharides such as mannitol, fucose, laminarin, alginate, fucoidan, and others (49, 50). Seaweeds also release components of dissolved organic nitrogen, including amino acids (51, 52). These algal hosts concentrate elements relative to seawater, including iron (53) and iodine (54, 55), elements that serve as enzyme cofactors needed by bacteria. Natural selection might act on bacteria to promote metabolic pathways that benefit the host (56), and these could include the provision of B vitamins that are typically not synthesized by eukaryotic hosts (11, 57). Bacterial residence on algal surfaces may also incur costs, mainly due to reactive oxygen species that are present on algal surfaces (58). We hypothesized that selection would act on host-associated bacteria to favor pathways that neutralize reactive oxygen species (ROS).

Here, we assessed whether genome properties and their encoded functional capacity differ in bacteria living in association with seaweed hosts versus free-living bacteria found in seawater. Across seaweed hosts, we identified (i) associated bacterial taxa that have been cultured and isolated, with their genomes sequenced either directly (whole-genome sequencing, WGS) or (ii) bacterial taxa inferred through an assembly of shotgun sequences (metagenome-assembled genomes, MAGs). We built a database of paired genera where a genome from a seaweed-associated bacterium was paired with that of a free-living congener, using published studies or genome repositories. We tested whether genome features, including genome size, GC content, and gene number, differed between host and free-living counterparts. We further examined metabolic features of both host-associated and free-living bacteria based on the predicted metabolic pathways encoded by their genes. Our pairing allowed us to determine if there was any evidence that natural selection has acted on the genomes of seaweed-associated bacteria in a consistent manner. Finally, we tested the complementarity of metabolisms between the brown alga *Ectocarpus subulatus* and its 28 bacterial isolates that all have sequenced genomes.

## MATERIALS AND METHODS

We contrasted the genomes of seaweed-associated versus free-living bacteria in seawater by pairwise comparison of bacterial taxa. Although the isolation and genome sequences of bacteria associated with seaweed are still a nascent area of research, there are an increasing number of genomes for analyses (35, 36, 59–61). We compared the genomes of bacteria isolated from seaweed to free-living bacteria, contrasting metabolic pathways at the genus level to reduce biases due to phylogeny. Bacteria isolated from seaweed were identified through several searches. First, we selected bacterial taxa recently isolated and sequenced by the authors (35, 36, 59, 60), including some bacteria isolated previously (61) for which genomes are reported here for the first time (Table S1). The methods for isolating these bacterial taxa and generating whole genome sequences are given in the citations. Second, we searched Bac*Dive* (https://bacdive.dsmz.de/, release 20.02.2023 [62, 63]) using the terms “algae,” “kelp,” and “seaweed.” DNA sequencing led to two types of genome information: either whole genome sequences from isolates (WGS) or MAGs from metagenome sequencing. Figure S1 shows our workflow, and Table S1 provides details on the origin of each taxon.

To build a collection of host-associated taxa paired with free-living taxa from seawater, we used online databases. Once we identified a taxon associated with a seaweed host, we searched for genome sequences of a congener in seawater using the online database Bac*Dive*. With the Advanced Search option, we used the criteria “genus”=Genus & “sampletype/isolated from”=seawater and selected strains with assembled genomes. If there were multiple seawater strains of the same genus in the database, we picked the first one on the list if there was only a single host-associated strain from that genus. In the case of multiple host-associated bacterial strains within a single genus, we searched for multiple free-living seawater strains in that genus. When no corresponding number of free-living strains in seawater was found, we randomly excluded one or several host-associated strains to maintain a balanced and fully paired data set. We chose WGS data over MAGs whenever possible. A second source of sequence data for free-living bacterial taxa was the TARA oceans database (https://fondationtaraocean.org/en/expedition/tara-oceans/) from which MAGs have been assembled for seawater microbes (6). We included MAGs when completion was at least 50% and redundancy was 10% or less (64). We refer to these genomes found in seawater through this search as “free-living,” although we recognize that they might have been in association with phytoplankton or other water column eukaryotes. Although the genomes are structured in pairs at the genus level to reduce phylogenetic effects, the true evolutionary relatedness of the matched genomes was unknown. Additionally, free-living bacterial genomes may have originated from an area geographically distinct from the seaweed host. For all strains, we started with files in the fasta format and quantified the features of the genome by running them through an identical pipeline.

We tested whether there were pronounced differences in the genomes of seaweed-associated bacteria versus those in the water column. The DNA sequences of all 144 genomes (72 pairs) were analyzed for genome features, including genome size, the number of genes, and GC content. We also tested for any biases in our comparisons by quantifying genome completeness. Using the anvi’o pipeline (version 7.1 [65]), we combined all fasta files into a standard format (anvi-script-reformat-fasta), generated a contigs database (anvi-gen-contigs-database), using Prodigal (66) to determine open reading frames for genes. Contrasts in genome features between seaweed-associated and free-living genomes were quantified after generating features for each genome individually (anvi-display-contigs-stats) and included genome size and the number of open reading frames. We used “clc-sequence-program” from the clc assembly cell tool (version 5.2.1) to determine GC content. We used mixed effects models to test for fixed effects of bacterial class and lifestyle, maintaining the paired structure of the data as a random effect (“lme4” in R, v1.1-36). There were four seaweed hosts that had 9–29 paired genomes, and we tested if genome patterns differed among these host species.

We tested whether there were significant differences in the predicted metabolic pathways of bacterial taxa that were seaweed-associated versus free-living taxa. We quantified the KEGG orthologs (anvi-run-kegg-kofams) and the presence of metabolic pathways in each genome (anvi-estimate-metabolism). Metabolic pathways described by metabolic modules were considered present if at least 75% of the KEGG genes (KEGG Orthologies, KOs) required to complete the metabolic pathway were identified (67), i.e., the default result in the anvi’o workflow. Our genome collection enabled a rigorous comparison of metabolic module similarity or distinctness as a function of seaweed-associated versus free-living (anvi-compute-metabolic-enrichment) (68). Our results were unchanged if we used 90% as a cutoff instead.

We analyzed whether taxonomy or lifestyle (seaweed-associated versus free-living) contributed to overall metabolic profiles with principal coordinate analyses (PCoA) based on Bray-Curtis distance. Metabolic modules with zero variance, either because the module was always present across all taxa or always absent, were removed from the analysis. PCoA was done with FactoMineR (version 2.9 [69]) and factoextra (version 1.0.7 [70]). R (version 4.3.1) packages and significance were tested with adonis2 (vegan, version 2.6.4 [71]).

Molecular functions across all 144 genomes were assessed by the presence or absence of KOs. We calculated the odds ratio (OR) for each ortholog as the number of genome pairs for which the KO term was present in the seaweed-associated genome and not in the seawater genome, divided by the number of genome pairs for which the KO term was present in the seawater genome and not in the seaweed-associated genome. Thus, KO terms more frequent in seaweed-associated bacteria had an OR of >1, while KO terms that were more prevalent in the free living had an OR < 1. An offset of 0.5 was added to the number of discordant pairs to prevent dividing by zero. Because different genome pairs could be formed if multiple genomes were present for a single genus, the entire procedure was repeated 100 times, generating randomly paired seawater and seaweed-associated genomes within the same genus. The median OR from the permutations was retained. We present the KO terms with extreme OR that were in the 95th or 5th percentile across all bacteria in the 5,914 total KO terms. Notable differences among *Flavobacteriia* KOs in their OR were examined for the 99th and 1st percentiles. When possible, we assigned KO terms to metabolic modules. KO analyses were done with R (version 4.3.1), ggplot2 (version 3.4.4), and dplyr (version 1.1.3).

We tested whether bacteria showed increased metabolic complementarity when associated with seaweed compared to free-living bacteria using metabolic networks as a predictor of potential metabolic interactions (37, 72). Here, *Ectocarpus subulatus* strain Bft15b was the only host for which a genome (34) and metabolic network (73) were publicly available, and we focused on quantifying metabolic complementarity in this holobiont. We annotated 28 genomes from bacteria associated with *Ectocarpus subulatus* and 28 corresponding free-living bacterial genomes, using Prokka version 1.14.6 (74). The resulting GenBank files were used to generate metabolic networks using Pathway Tools version 26.0 (75) and the mpwt wrapper implemented in Metage2metabo version 1.6.0 (76). The host metabolic network was taken from reference 73, and the composition of the culture medium in Provasoli-enriched seawater was based on reference 77. Metabolic scopes, i.e., metabolites that can be produced from compounds available in their culture media, were calculated individually for the host metabolic network, each bacterial metabolic network, and each bacterial metabolic network merged with *E. subulatus* using MisCoTo version 3.2.0 (72). The “added value” of each metabolic cooperation between the host and each bacterium was defined as the metabolites produced by the merged network, synthesized by neither the host nor the bacterial network alone. These values were then compared between *Ectocarpus*-associated and free-living bacteria using binomial tests with correction for false discovery rate. The two-way ANOVA was carried out using Past version 4.02 (78).

## RESULTS

We found 72 genomes across 16 seaweed hosts that spanned brown (Phaeophyceae), red (Rhodophyceae), and green (Chlorophyceae) divisions of macroalgae (Table 1; Fig. S2) that had corresponding free-living congeners from seawater for a total of 144 bacterial genomes (Table S1). The selected seaweed-associated genomes were overwhelmingly from brown algal hosts (89%). *Ascophyllum nodosum* was the host of 13 genomes (60) (Table S1), while 23 whole genome sequences came from *Ectocarpus subulatus* (35), with another 5 MAGs (36). There were nine isolates (61) with WGSs from the kelp *Laminaria digitata* (Table S1), and nine WGSs from the kelp *Nereocystis luetkeana* (59), with a single MAG (79). Four additional WGSs were isolated from seaweeds in Corsica, using the methodology described in reference 80. Overall, 66 of 72 whole genome sequences resulted from efforts to isolate bacteria directly from seaweeds.

**TABLE 1:** Host seaweed species with the number of bacterial taxa associated with each^*a*^

Host species	Seaweed class	Number of isolates
*Acrosiphonia sonderi*	Chlorophyceae	1
*Ascophyllum nodosum*	Phaeophyceae	13
*Corallina officinalis*	Rhodophyceae	1
“Corallinales”	Rhodophyceae	1
*Cutleria multifida*	Phaeophyceae	1
*Cystoseira* sp*.*	Phaeophyceae	1
*Ectocarpus subulatus*	Phaeophyceae	28
*Eucheuma* sp*.*	Rhodophyceae	1
*Halopythis incurva*	Rhodophyceae	1
*Jania* sp*.*	Rhodophyceae	1
*Laminaria digitata*	Phaeophyceae	9
*Nereocystis luetkeana*	Phaeophyceae	10
*Porphyrayezoensis*	Rhodophyceae	1
*Sargassum* sp*.*	Phaeophyceae	1
*Sebdenia* sp*.*	Rhodophyceae	1
*Undaria* sp*.*	Phaeophyceae	1
		72

^*a*^ All taxa were identified to genus or species level except a representative of the red alga order Corallinales.

The 72 bacterial genomes from seaweed hosts were diverse and represented 35 different genera across 3 phyla and 4 total classes (Table S1). The majority (40) belonged to the phylum *Pseudomonadota*, with 8 *Alphaproteobacteria* and 32 *Gammaproteobacteria*. The phylum *Bacteroidota* was represented by 29 genomes, all class *Flavobacteriia*; the remaining 3 genomes were in the *Actinomycetota,* and all were in the class *Actinomycetia*. Across seaweed-associated genomes, only 6 were MAGs (*Granulosicoccus, Maribacter,* and *Marinobacter*), while 66 were whole genome sequences from isolates. In the free-living counterparts, 11 of the 72 genomes were inferred from MAGs. Selected bacterial taxa differed among algal hosts, with the brown alga *Ectocarpus* having the greatest diversity of associated bacteria. In contrast, the sequencing efforts for bacterial strains associated with *Ascophyllum* targeted *Flavobacteriaceae* only (Fig. S2).

### Features of host-associated versus free-living genomes

**Fig 1:**
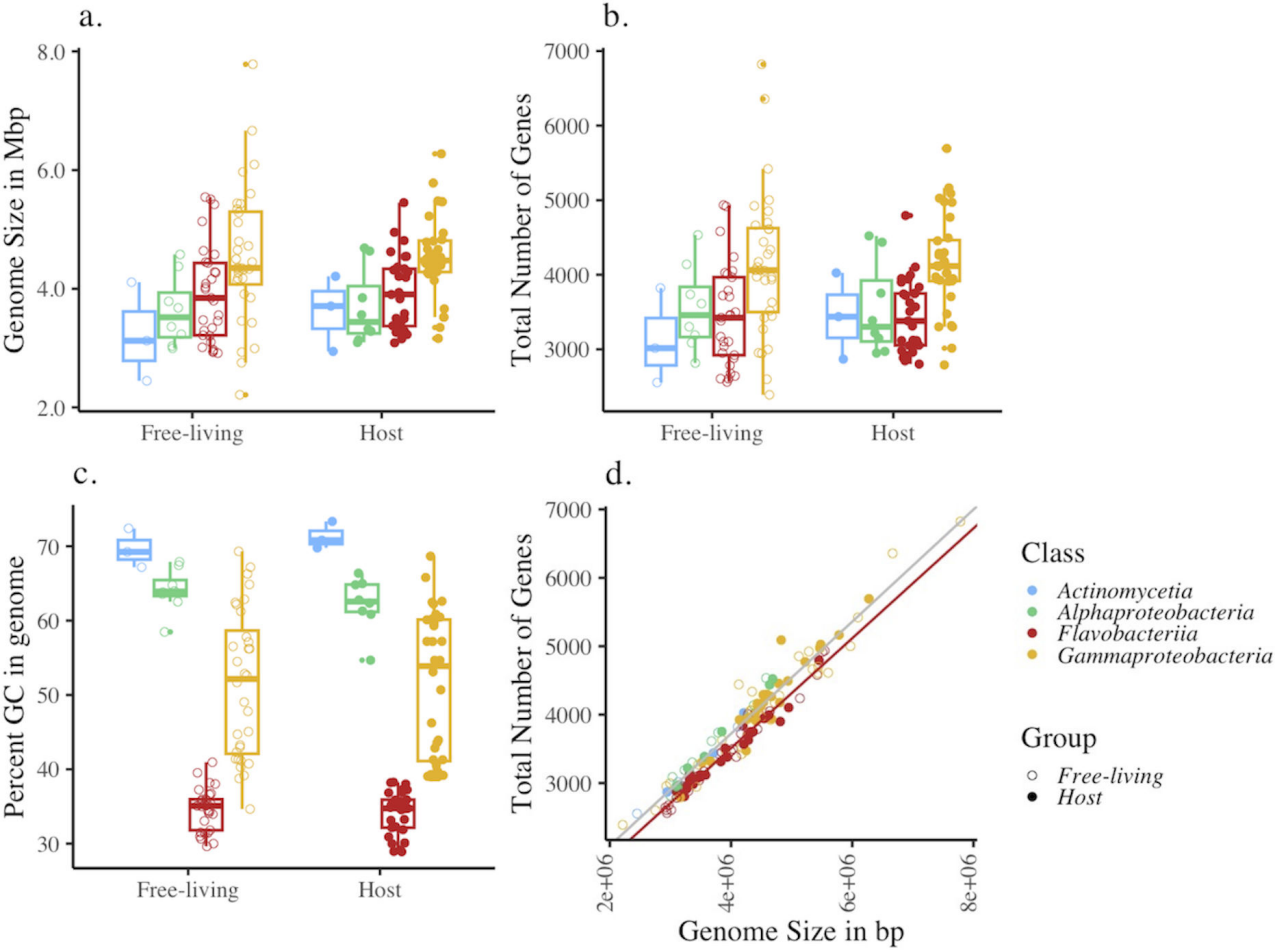
Features of the genomes within four bacterial classes used in this study, consisting of 72 pairs of genera that were matched as host-associated on macroalgae or free-living in seawater, where (a) genome size in bp, (b) the total number of genes identified by Prodigal, (c) the percentage of the genome that was GC, and (d) the relationship between the number of genes and the genome size. There were no differences between seaweed-associated and free-living taxa in panels a–c, though genome size and the total number of genes were greatest for *Gammaproteobacteria* (*P* = 0.002 and *P* = 0.020, respectively, Tables 2 and 3). The GC content was distinct for *Flavobacteriia* and *Gammaproteobacteria* (*P* < 0.001, Table 4). The total number of genes to genome size was lower for *Flavobacteriia* compared to the other classes of bacteria (Table 6). The red line is the regression line for *Flavobacteriia*; the gray line is for the other three classes.

When we compared all bacterial pairs, the genome size, gene number, and GC content remained unchanged regardless of whether taxa were seaweed-associated or free-living, though there were some differences at the host level. Taxonomy, however, was a strong determinant of all three genomic features (Fig. 1a through c). Genome size varied over threefold across the bacterial taxa we analyzed, from a minimum of 2.21 million base pairs to 7.78 million, with a mean of 4.15 million. Seaweed-associated taxa averaged 4.14 million in length, while free-living taxa averaged 4.17 million. The taxonomic affiliation was the greatest explanatory variable (Fig. 1), even when we treated pairs as random variables in statistical analyses (Tables 2 to 6). *Gammaproteobacteria*, though having the smallest and the largest genome, had a significantly greater average genome size than the other three classes of *Actinomycetia*, *Alphaproteobacteria*, and *Flavobacteriia* (Fig. 1a). A model that included whether taxa were host-associated did not explain the added variation in the data (*P* = 0.852, parametric bootstrap model comparison, Table 2, Fig. 1a). However, when we analyzed the bacteria associated with each brown algal host as a separate group, *N. luetkeana*-associated bacterial taxa had a smaller genome than their free-living counterparts (4.43 versus 5.10 million base pairs, respectively, Table S2; Fig. S3).

**TABLE 2:** A linear mixed effects model testing the role of bacterial class and living mode on the genome size, with pair structure as a random effect^*a*^

Fixed effects	Estimate	SE	df	*t*-value	Probability	
(Intercept)	3,411,407	341,605	70.8	9.986	<0.001	***
ClassAlphaproteobacteria	239,261	392,474	68	0.610	0.543	
ClassFlavobacteriia	500,759	351,589	68	1.424	0.157	
ClassGammaproteobacteria	1,128,975	350,041	68	3.225	0.002	**
GroupHost	25,603	136,642	71	0.187	0.852	

^*a*^ The inclusion of living mode did not improve model fit when a model with and without living mode was compared with a parametric bootstrap (1,000 samples, *P* = 0.775, “Pbmodcomp”). *Gammaproteobacteria* had the greatest mean genome size. *** denotes *P* < 0.001; ** denotes 0.001 > *P* < 0.01.

**TABLE 3:** A linear mixed effects model testing the role of bacterial class and living mode on the total number of genes, with pair structure as a random effect^*a*^

Fixed effects	Estimate	SE	df	*t*-value	Probability	
(Intercept)	3,281.1	347.1	70.3	9.453	<0.001	***
ClassAlphaproteobacteria	263.2	403.7	68	0.652	0.517	
ClassFlavobacteriia	162.6	361.6	68	0.450	0.654	
ClassGammaproteobacteria	857.3	360	68	2.381	0.020	*
GroupHost	13.2	88.8	71	0.149	0.882	

^*a*^ The inclusion of living mode did not improve model fit when a model with and without living mode was compared with a parametric bootstrap (1,000 samples, *P* = 0.761, “Pbmodcomp”). *Gammaproteobacteria* had the greatest mean gene number. *** denotes *P* < 0.001; * denotes 0.001 > *P* < 0.05.

**TABLE 4:** A linear mixed effects model testing the role of bacterial class and living mode on the GC content, with pair structure as a random effect^*a*^

Fixed effects	Estimate	SE	df	*t*-value	Probability	
(Intercept)	70.7	3.9	68.3	17.899	<0.001	***
ClassAlphaproteobacteria	−7.3	4.6	68	−1.579	0.119	
ClassFlavobacteriia	−36.1	4.1	68	−8.72	<0.001	***
ClassGammaproteobacteria	−19.1	4.1	68	−4.639	<0.001	***
GroupHost	−0.4	0.4	71	−0.926	0.357	

^*a*^ The inclusion of living mode did not improve model fit when a model with and without living mode was compared with a parametric bootstrap (1,000 samples, *P* = 0.450, “Pbmodcomp”). *Gammaproteobacteria* had the greatest mean GC content, and *Flavobacteriia* had a significantly smaller GC content. *** denotes *P* < 0.001.

**TABLE 5:** Analysis of the percentage of GC content as a function of the total length of the genome^*a*^

Fixed effects	Estimate	SE	df	*t-*value	Probability	
(Intercept)	71.9	4.2	83.3	17.16	<0.001	***
total_length	0	0	88.6	−0.9	0.371	
ClassAlphaproteobacteria	−7.2	4.6	68	−1.56	0.123	
ClassFlavobacteriia	−35.9	4.1	68.3	−8.668	<0.001	***
ClassGammaproteobacteria	−18.7	4.1	69.5	−4.51	<0.001	***
GroupHost	−0.4	0.4	70.2	−0.9	0.371	

^*a*^ With a linear mixed effects model or traditional linear model, there was no effect of whether bacteria are host-associated or free-living on the percentage of GC content. *** denotes *P* < 0.001.

**TABLE 6:** The number of genes as a function of genome length by class^*a*^

Fixed effects	Estimate	SE	df	*t*-value	Probability	
(Intercept)	462.8	97.3	95.6	4.758	<0.001	***
total_length	0	0	136.3	47.811	<0.001	***
ClassAlphaproteobacteria	65.6	89.8	66.9	0.730	0.468	
ClassFlavobacteriia	−251.1	80.8	67.5	−3.107	0.003	**
ClassGammaproteobacteria	−75.4	82.3	70.3	−0.915	0.363	
GroupHost	−7.9	23.2	69.9	−0.343	0.733	

^*a*^ With a linear mixed effects model or regular linear model, there is no effect of whether bacteria are host-associated or free-living on the slope, e.g., lmer(Num_Genes_prodigal ~ total_length + Class + (1 | pair_code), data = genome_data). *** denotes *P* < 0.001; ** denotes 0.001 > *P* < 0.01.

The number of genes within each genome followed a similar trend to that of genome size (Fig. 1b), with taxonomy being a strong explanatory factor and *Gammaproteobacteria* having the greatest mean number of genes at 4,145 (Table 3). Across all genomes, the mean gene number was 3,763, with 3,770 for host-associated taxa and 3,757 for free-living taxa. A model that included whether taxa were host-associated did not explain gene number (*P* = 0.882, parametric bootstrap model comparison, Table 3, Fig. 1b).

GC content varied over twofold from 28.9% to 73.4% with a mean of 46.6%. The means were nearly identical across the two groups and all hosts, with genomes of bacteria isolated from seawater showing 46.8% GC, while genomes from seaweed-associated bacteria were 46.4% GC. However, *A. nodosum*-hosted bacterial genomes differed in GC content compared with free-living counterparts, with decreased GC content in association with the host. Overall, taxonomy was a strong explanatory variable for GC content (*P* < 0.001, Fig. 1c). *Actinomycetia* and *Alphaproteobacteria* had the highest GC content with means of 70.5% and 63.2%, respectively, followed by *Gammaproteobacteria* (51.3%), and *Flavobacteriia* at the lowest with 34.4%. A model that included whether taxa were host-associated did not explain variation in GC content (*P* = 0.357, parametric bootstrap model comparison, Table 4, Fig. 1c). The pattern of relatively high GC content in the *Actinomycetia* and *Alphaproteobacteria* remained when we normalized to genome size (Table 5).

Across all genomes, the ratio of gene number to genome size did not differ based on whether a genome was host-associated or free-living (*P* = 0.733, Fig. 1d, Table 6). *Flavobacteriia* did, however, differ from the other three classes of bacteria, showing a lower number of genes per genome length (*P* = 0.003, Table 6; Fig. 1d).

### Metabolic features of host-associated versus free-living genomes

**Fig 2:**
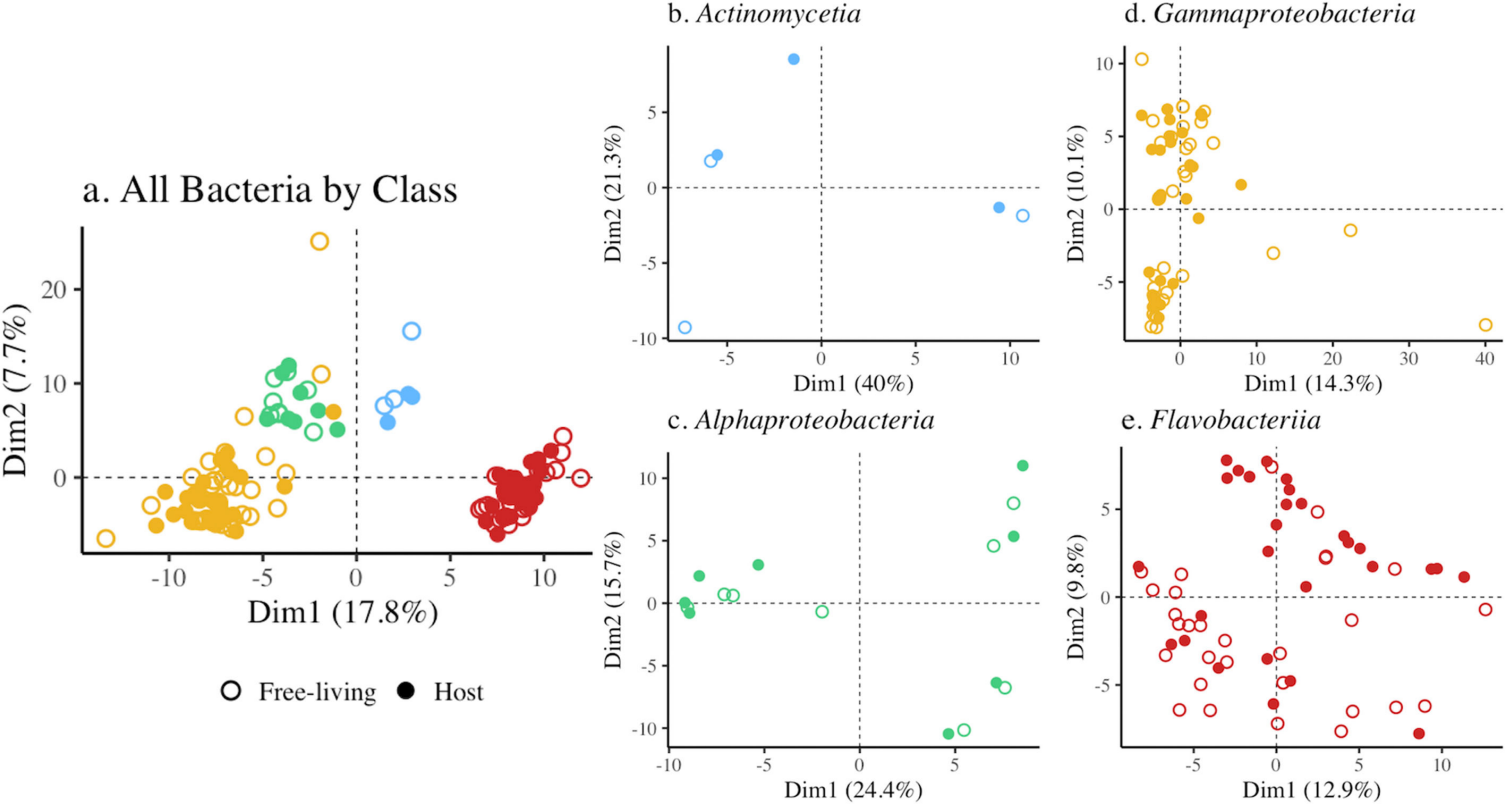
Principal coordinate analysis of the completion of metabolic modules (Table S4) for (a) all 144 genomes, where samples are represented by color by bacterial class and symbol type for seaweed-associated versus seawater (72 pairs). There were no overall metabolic differences between bacteria that were seaweed-associated versus from seawater, but taxonomic class was highly significant (Table S3, Adonis with 999 permutations based on Bray-Curtis distances). Data separated by class showed no statistical difference for seaweed-associated versus free-living for (b) *Actinomycetia*, (c) *Alphaproteobacteria*, or (d) *Gammaproteobacteria*. Only class *Flavobacteriia* (e) showed a distinction in metabolism between seaweed-associated and free-living (*P* = 0.016). The strong effect of phylogenetic history extended to the order level of bacteria too (*P* < 0.001).

Bacterial taxonomy was the main determinant of metabolic capabilities inferred from bacterial genomes, regardless of whether the bacterial taxon was seaweed-associated or isolated from seawater. When all taxa were included, taxon pairs clustered in multivariate space, and PCoA analyses indicated no separation between seaweed-associated bacteria or those isolated from seawater but did indicate significant differences at the level of class (Fig. 2, *P* = 0.001, Table S3a and b) or order (*P* = 0.001, Table S3g). Similarly, metabolic features of host-associated and free-living taxa pairs were indistinguishable for class *Actinomycetia* (*P* = 0.800, Table S3c), and the proteobacterial classes of *Alphaproteobacteria* (*P* = 0.943, Table S2d), and *Gammaproteobacteria* (*P* = 0.833, Table S3e). Within the class *Flavobacteriia*, however, seaweed-associated and free-living bacteria had statistically distinct metabolic pathways (Fig. 2, *P* = 0.016, Table S3f).

Although metabolic module completeness varied across genomes and modules (Table S4), metabolism showed little difference between host-associated and free-living bacteria based on the analysis of metabolic module completeness with anvi’o (Table S5). When combined across all host taxa, there were no statistically significant differences once corrections for multiple comparisons were made (Table S5b). Even when results were separated by class, there was no statistical difference in the metabolic module completeness in *Actinomycetia*, *Alphaproteobacteria*, *Gammaproteobacteria*, and *Flavobacteriia* when corrected for multiple tests. However, some modules had suggested differences, particularly in the *Flavobacteriia*. The paired structure of genomes, however, was not maintained in these group analyses. When the paired structure was maintained with binomial tests and a Benjamini-Hochberg correction, seven modules were significantly enriched in the free-living bacteria compared to host-associated ones (Table S5a): two modules for Lysine biosynthesis (M00030 and M00433), trans-cinnamate degradation (M00545), sulfate-sulfur assimilation (M00616), Pimeloyl-ACP biosynthesis (M00572), Ethylmalonyl pathway (M00373), UDP-N-acetyl-D-glucosamine biosynthesis (M00892), and the Semi-phosphorylative Entner-Doudoroff pathway (M00308). Furthermore, two modules related to D-Galacturonate degradation (M00631 and M00552) were significantly more complete in the host-associated genomes (Table S5a).

### Genes that differed in occurrence between host-associated and free-living taxa

**Fig 3:**
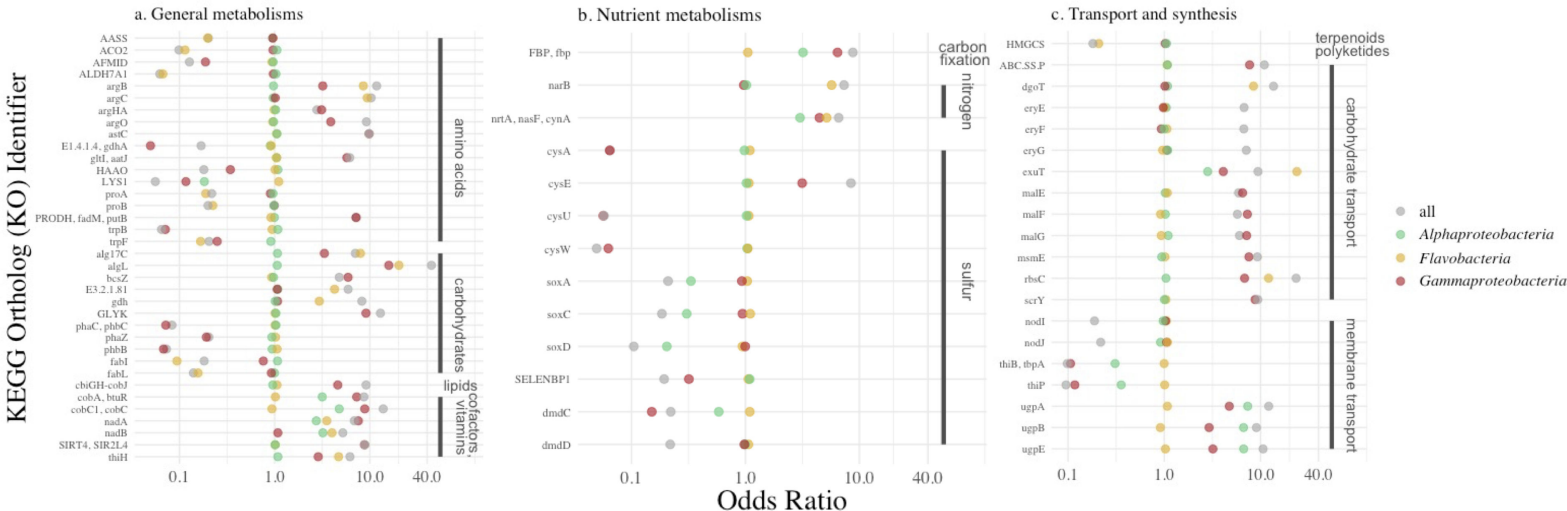
The occurrence of genes in bacterial taxa that showed distinction among seaweed-associated versus free-living genomes, grouped into (a) general metabolisms, (b) nutrient metabolisms, and (c) transport and synthesis functions. All values are based on the odds ratio (Table S6). Only genes in the 1% tails of all OR values are shown for the three most abundant classes of bacteria and all bacterial taxa together. Values > 1 are enriched in host-associated bacteria, while values < 1 are more prevalent in free-living bacterial genomes. The metabolic processes that are suggested to differ the most are shown in Fig. 4.

**Fig 4:**
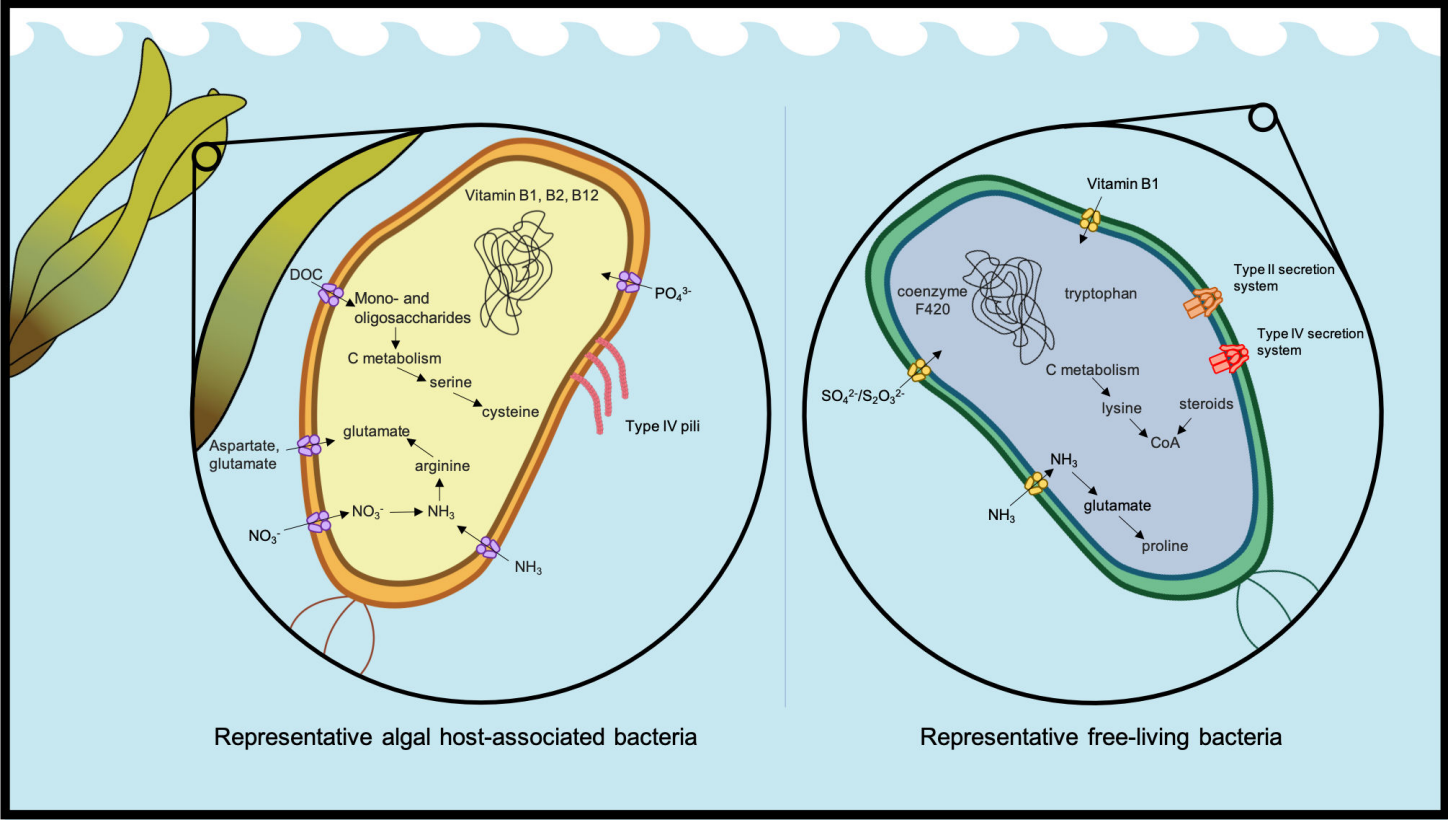
A schematic of the bacterial metabolisms that differed between seaweed-associated (left) and free-living (right) based on analyses of KEGG Ortholog genes (Fig. 3; Table S6).

A paired analysis of the occurrence of genes in bacterial taxa that were seaweed-associated versus free-living showed differences in metabolic features and processes. When ORs in the 5th and 95th percentiles of all OR were considered, 380 KO terms were enriched in host-associated bacterial genomes and 297 in free-living genomes (Table S6; Fig. 3). Thirty-seven percentage of KO terms had an OR of 1.0 (2,201 out of 5,914), and many more were only weakly affiliated with host-associated or free-living (interquartile range of 0.74–1.65). An illustration of the processes most affected by the enrichment or depletion of different KEGG genes is shown in Fig. 4.

Seaweed-associated bacteria showed a trend toward greater B vitamin production capacity, including cobalamin biosynthesis (vitamin B12; K02225, *cobC1* OR = 13.0; K19221, *cobA* OR = 9.0; and K13541, *cobJ* OR = 9.0), thiamine (K03150, *thiH* OR = 5.8), and niacin (B3) related compounds, including nicotinate (K11414 *Sir2L4* OR = 9) and nicotinate D-ribonucleotide (K00278, *nadB* OR = 5.0; K03517, *nadA* = 6.3). In contrast, free-living bacteria showed enrichment for thiamin import (K02064, *thiB* OR = 0.11; K02063 *thiP* OR = 0.11) but not synthesis (Fig. 3a; Table S6).

Seaweed-associated bacteria were enriched in genes involved in the breakdown of algal exudates and cell wall polysaccharides, including alginate and agar breakdown (*algL*, OR = 41.0; *alg17C*, OR = 6.17; and beta-agarase, OR = 6.0, Fig. 3a). Genes that aid mono- or oligosaccharide metabolism and transport (*eryG/eryF/eryE; ugpB/ugpA/ugpE; rbsC; dgoT; msmE; scrY; exuT; malE/malF/malG, bcsZ, kdgK, iduronate-2-sulfatase, cycB, lplA/lplB/lplC*), including for glucose and fructose (*gdh* and *fbp*), were also enriched in seaweed-associated bacteria (Fig. 3a and c; Table S6).

Some differences in amino acid metabolism and synthesis occurred between seaweed-associated and free-living bacteria. Free-living bacteria tended to have more genes required for the biosynthesis of tryptophan (*trpB* and *trpF*) and lysine (*ACO2* and *Lys1*). Both groups were enriched in different amino acid metabolic pathways and membrane transport proteins, though only host-associated bacteria had an increase in transport related to glutamate and aspartate. In free-living bacteria, increased metabolic capability related to glutamate was suggested by enriched dehydrogenase genes, including *gdhA*, and *proA* and *proB* genes from which glutamate can be metabolized to ornithine, then arginine/putrescine and acetyl CoA, or redirected to amino acid synthesis (Fig. 3a; Table S6).

Nitrate uptake and transformation to ammonium were enhanced in seaweed-associated bacteria based on *nrtA* (K15576, OR = 5.4) and *narB* (K00367, OR = 6, Fig. 3b). Arginine biosynthesis is heavily overrepresented in seaweed-associated bacteria (K14681, *argH* OR = 11.0; from glutamate K00930, *argB* OR = 11.0; K00145, *argC* OR = 11.0; K22477, *argO* OR = 9.0, Fig. 3a). Other substrates such as proline could be used for arginine biosynthesis (K00318, PRODH OR = 7.0). Arginine, in turn, can be converted back to glutamate (K00840, *astC* OR = 9.0).

Free-living bacteria showed differences in carbon and protein metabolism compared with seaweed-associated bacteria, with perhaps a greater role for acetyl-CoA (Table S6). Amino acid pathways linked to CoA synthesis, such as lysine degradation pathways (α-aminoadipic semialdehyde synthase and aldehyde dehydrogenase 7 family, member A1 [ALDH7A1]), were more complete in the genomes of free-living bacteria (Fig. 3a). Phenylalanine and more pathways involving tryptophan (AFMID and HAAO) were also present in the genomes of free-living bacteria. There was also a higher potential to produce fatty acids from aldehyde (K14085) and pyruvate via acetyl-CoA (*phaZ, phaC, phbB*, HMGCS, *fabI,* and *fabL*) in free-living bacteria. The genomes of free-living bacteria also showed enhanced lipo-oligosaccharide importers (*nodJ* and *nodI*, Fig. 3c).

Genes related to sulfur metabolism differed between the two groups of bacteria (Fig. 3b). Free-living bacteria had increased function for extracellular sulfur uptake via the genes *cysU* (OR = 0.05), *cysW,* and *cysA* (OR = 0.05). Sulfate production from thiosulfate (*soxA, soxC,* and *soxD*) and dimethylsulfoniopropionate (DMSP) synthesis from sulfate (*dmdC, dmdD*, and SELENBP1) were also present in higher frequency in free-living bacteria. In contrast, sulfur-related functions in seaweed-associated bacteria showed a higher frequency of genes involved in the synthesis of serine (from glycerate; K15918, OR = 13.0), as well as the synthesis of cysteine from serine (K00640, *cysE*, OR = 9.0).

The type II secretion system (*gsp* D/J OR = 0.1; *gsp* F/G/K/L/N OR = 0.2) and the type IV secretion system (*vir* B2/B3/4/9/10 OR = 0.2) were more frequently observed in free-living bacteria. Host-associated bacteria were enriched in genes related to type IV pili (*msh A/B/C/D/E/I/J/N/O/P,* and *pilJ/Q/V/X*) (Table S6).

Among all bacterial classes, *Flavobacteriia* showed the greatest differences in metabolic capabilities between a host-associated and free-living lifestyle (Fig. 2). When we compared KEGG enrichment within the *Flavobacteriia* only and in the upper and lower 1% of the distribution of OR, host-associated bacteria showed enrichment in nitrate and nitrite transporters (*nrtA,B, nasE,F,* Fig. 3b; Table S7). The *mtlK* gene for metabolizing mannitol was also enriched in host-associated *Flavobacteriia*, as were genes related to the amino acid metabolism of glutamine and asparagine, and potentially related to alginate metabolism (*algL, alg17c, kduD, kdgK,* and *eda*).

### A test of metabolic complementarity with the brown alga *Ectocarpus*

**Fig 5:**
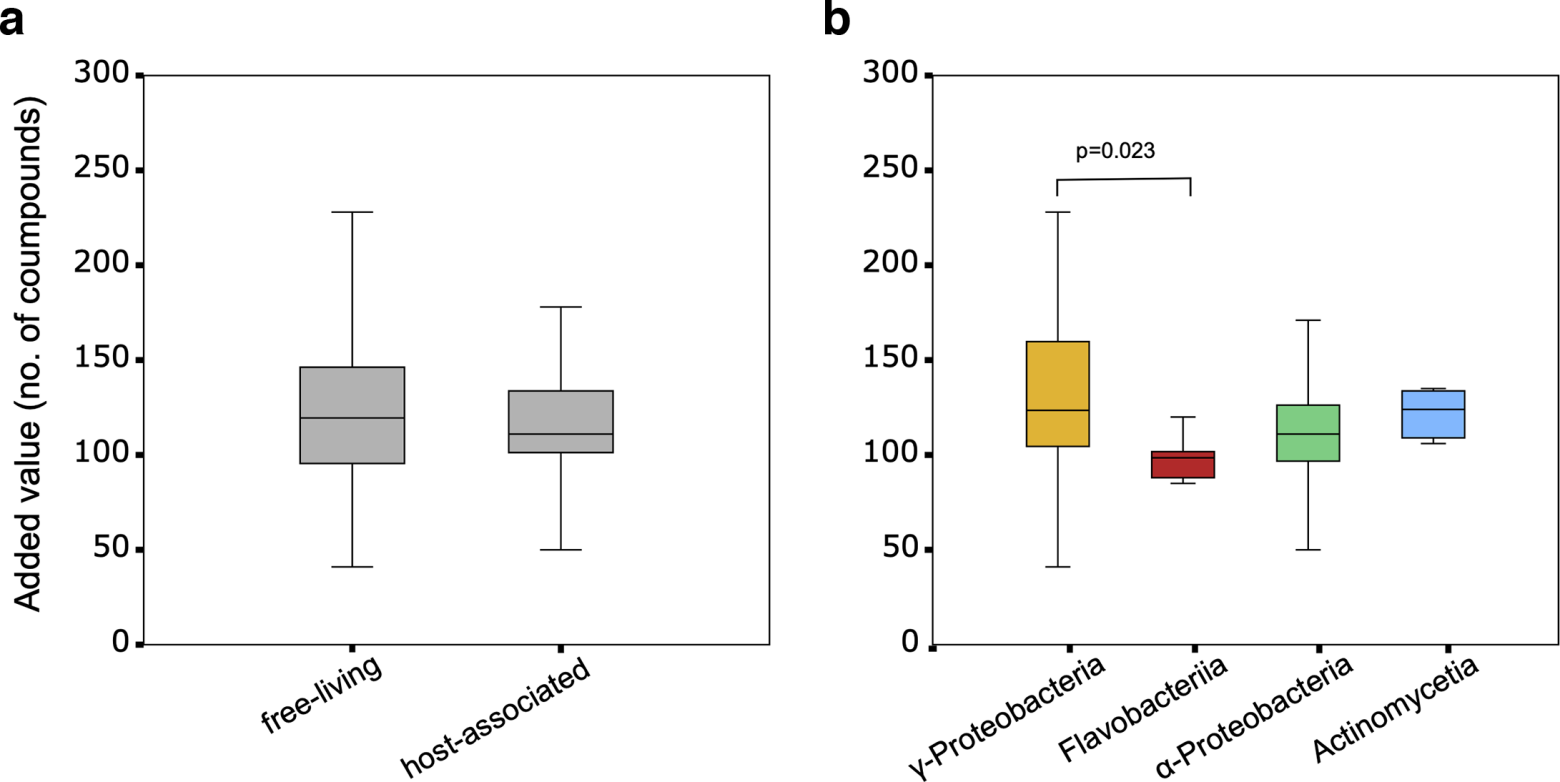
(a) Metabolic complementarity as estimated by the number of “added value” compounds producible only via the cooperation with the host seaweed *Ectocarpus subulatus* (Table S8), estimated in both seaweed-associated versus free-living bacteria (*n* = 28 in both cases; Table S1). (b) Metabolic complementarity as estimated by the number of “added value” compounds producible only via the cooperation with the seaweed host across different bacterial classes. The *P*-value was obtained from a Tukey’s HSD test after a two-way ANOVA (*P* for class = 0.048). The data set comprises 4 *Actinomycetia*, 12 *Alphaproteobacteria*, 10 *Flavobacteriia*, and 30 *Gammaproteobacteria*. Means and standard deviations are shown.

A total of 466 added-value compounds were identified across all analyses of the *Ectocarpus* holobiont. The complete matrix of compounds for each host-bacterium pair can be found in Table S8. A direct comparison of the number of added-value compounds between host-associated and free-living bacteria revealed no significant difference. The average added value of host-associated bacteria was 119 (±29.9 SD) compounds versus 121 (±39.98 SD) for free-living bacteria (Fig. 5). On the one hand, the compound most biased toward the host-associated bacteria was dihydroxyacetone, which was part of the added value of eight host-associated bacteria versus two free-living bacteria. On the other hand, UDP-*N*-acetyl-alpha-D-mannosaminuronate was found five times in the added value of free-living bacteria but never in the added value of host-associated bacteria. However, none of these differences were statistically significant after correction for multiple testing. The strong effect of taxonomy on metabolic results that we quantified in other analyses was also observed here on the number of added-value compounds. A two-way ANOVA with “class,” “lifestyle,” and the interaction term “class*lifestyle” showed that only “class” was significant. Among the pairwise comparisons, *Flavobacteriia* solely had a significantly lower added value (97.6 ± 10.1) than *Gammaproteobacteria* (131 ± 39.5) (Fig. 5).

## DISCUSSION

### The genome size of host-associated versus free-living bacteria

Across all bacterial taxa, whether we classified a bacterium as seaweed-associated or free-living was not a strong determinant of genome size; rather, taxonomy was the greatest determinant of genome size, with the largest genome size found within the *Gammaproteobacteria*. The only difference in genome size occurred with those bacteria associated with *N. luetkeana*, where host-associated bacterial genomes were on average smaller in the 10 paired genomes of *Flavobacteriia* and *Gammaproteobacteria*. In a previous study of pelagic bacteria, only obligate symbionts or parasites had a reduced genome, while the genome size of host-associated taxa did not differ from free-living ones (81), analogous to the results reported here.

Many marine host-associated bacteria may have to spend part of their life cycle free living in seawater, and gene reduction may not be advantageous. The genomes of human gut bacteria that were good colonizers of the gastrointestinal tract were larger than those of poor colonizers (67), suggesting that a full suite of metabolic capabilities was retained in colonizing bacteria. While there have been several investigations of genome size among environments and with host association, the findings have not revealed strong patterns (82, 83). Although a relationship between reduced genome size and the transition to a beneficial endosymbiotic lifestyle exists for some well-studied microbial symbionts, such as the *Sodalis*-related endosymbiotic bacteria (*Gammaproteobacteria* and *Enterobacterales*) associated with insects (38), this relationship is less clear in other contexts, including this study.

### GC content

The genomes examined revealed that taxonomy was a determinant of GC content, while there was only a single instance where GC content was related to whether bacteria were host-associated or free-living (84). GC content was higher in free-living *Flavobacteriia* that were counterparts to those cultured from the brown alga *A. nodosum*. Although increased GC content in free-living bacteria has been suggested to be the result of natural selection on growth rate (85), beneficial endosymbionts are thought to evolve under weakened selection pressures, leading to AT-enriched genomes resulting from bacterial mutational bias, a lack of cellular machinery for recombination, and DNA mismatch repair systems limiting GC to AT substitutions (38). Our analyses suggest that higher order taxonomy was overwhelmingly important compared with lifestyle, with *Flavobacteriia* showing the smallest GC content, a result reported previously in marine *Bacteroidota* (86). GC content has also been connected to carbon use, particularly metabolic preferences for sugars versus amino and organic acids, and thus might be a determinant of “metabolic niche space” (45). Similarly, model investigations showed that the type of carbon compound available is associated with differences in GC content (87) and may account for host-specific patterns in GC, such as we saw in *A. nodosum* here. Although we do not know the precise resources that each host could provide to its bacterial associates, dissolved organic carbon from seaweeds is pervasive in coastal ecosystems (47) and elevated in proximity to seaweed beds (88). Macroalgal tissues are rich in carbon resources with a mean C:N of 20, a value much greater than phytoplankton (89). Seaweeds are the origin of a diversity of carbon-rich compounds, including monosaccharides such as glucose, galactose, mannose, xylose, rhamnose, fucose, and arabinose, and complex polysaccharides such as laminarin, fucoidan, alginate, carrageenan, and agar (90). Our results add to others in suggesting a relationship between low GC content, bacterial phylogeny, and the use of carbon compounds, including complex polysaccharides, though we recognize that more investigation is needed in this area.

### Metabolic capabilities

As with genome features such as size and GC content, evolutionary history was also a key explanatory factor for differences in metabolic capabilities. Our multivariate analyses indicated strong differences in metabolic modules among class- and order-level taxonomy (Fig. 2), a pattern seen in other community analyses (45). However, at the level of KEGG orthology, and when our analyses were paired, we detected pronounced differences in the frequency of genes present in either host-associated or free-living bacteria. Consistent with a lifestyle associated with seaweeds (91), many host-associated bacterial genomes were enriched in genes related to the use of simple and complex carbon compounds, including mannitol, a simple sugar almost exclusive to brown algae (92). *Flavobacteriia* and *Gammaproteobacteria*, known for polysaccharide utilization loci used for algal polysaccharide degradation (93), represent a major component of seaweed biofilms and were highlighted in our study. *Flavobacteriia* and *Gammaproteobacteria* also have the capability to metabolize alginate (94), an abundant resource also produced by brown algae (95). A study with terrestrial plant-associated bacteria showed an increase in metabolisms related to carbohydrates and their transport compared to related bacteria that were not associated with host plants (82), possibly highlighting a general phenomenon where carbon is a relative surplus provided by terrestrial and marine phototrophs that are limited by other elements (48, 96). Bacteria are adapted to use the byproducts.

Seaweed-associated bacteria had an increased metabolic capability for synthesizing B vitamins, including vitamin B12 (cobalamin). Seaweeds, like other eukaryotes, are likely unable to produce B vitamins (57, 97), and association with B vitamin-producing bacteria may be an important means of obtaining these essential micronutrients (11). For example, in ocean plankton, the provision of vitamin B12 from bacteria to diatoms has been appreciated for decades (98). Not all bacteria associated with seaweeds produce any or all B vitamins (59), suggesting that auxotrophic relationships may develop in biofilms on seaweeds. Vitamin B4, choline, and its precursor betaine are osmoprotectants (99, 100), and increased betaine metabolism in host-associated bacteria could be related to osmoprotection. Overall, the metabolism of B vitamins is strongly associated with host-dwelling microbes and may be a common currency for positive species interactions in the ocean (101, 102). Whether B vitamins underpin seaweed-bacteria interactions is an area of research that deserves more empirical effort.

Host-associated and free-living bacterial genomes indicated differences in amino acid metabolism. Seaweed-associated bacterial genomes indicated enrichment in genes related to the transport of glutamine and asparagine, while free-living bacteria also had metabolic capability for glutamate transport. Glutamate and aspartate have been shown to be amino acids with the greatest concentration in seawater (103) and many seaweeds (104, 105) and also contain two nitrogen atoms. Furthermore, microbes associated with the kelp *Nereocystis luetkeana* grew best in amino acid-enriched media that contained these two amino acids (59). Seaweed-associated bacteria appear to be able to both synthesize and break down glutamate. Arginine is also an important component of seaweeds (104), and genes for its metabolism were better represented in host-associated bacteria. In contrast, free-living bacteria were characterized by an increased presence of biosynthetic genes for tryptophan, proline, and lysine, perhaps reflecting scarcer amino acid resources in the water column.

Algal interactions involving sulfur are important in coastal environments with consequences to Earth’s biogeochemical cycles (106). Therefore, bacterial interactions with seaweed that are mediated through or related to sulfur are highly relevant. The synthesis of cysteine from serine was enriched in seaweed-associated bacteria, suggesting that these amino acids could be a bacterial sulfur source for the seaweeds. Sulfur-containing metabolites, including cystine and cysteine, mediate reactive oxygen species in *Escherichia coli* (107) and may be equally relevant in other bacteria. Cysteine metabolism from serine was enriched in seaweed-associated bacteria, though sulfo-cysteine was equally present in both free-living and host-associated bacteria with *Ectocarpus subulatus*, and both free-living and host-associated bacteria had multiple sulfur metabolisms (Table S6). Free-living bacteria had a diversity of metabolic pathways for sulfur acquisition, including *dmdC* genes*, dmdD* genes, and others (Fig. 3b; Table S6), perhaps indicating that sulfur acquisition requires a diversity of pathways for bacteria that are not in association with seaweed hosts. The prevalence of algal polysaccharides that are sulfated (108) and the bacterial degradation pathways capable of desulfation (109) may indicate that seaweed-associated bacteria have increased access to sulfate when degrading algal polysaccharides. Bacterial and algal interactions with sulfur may also occur through dimethylsulfoniopropionate, an important organosulfur compound in the coastal ocean and produced by macrophytes and phytoplankton (58, 110). Bacteria degrade DMSP to volatile dimethyl sulfide (DMS), and kelp-associated bacteria have the gene *dddp* to catalyze the conversion of DMSP into DMS (59), which, in turn, is able to neutralize HO• (111). Seaweed-associated bacteria in our study had enhanced dimethyl sulfoxide reductase (*dmsA, B,* and *D*), which could play a role in ROS detoxification. The role of DMS as an “anti-greenhouse gas” (112) and the importance of ROS in climate-related stress for coastal species highlight the need for a deeper understanding of DMSP cycling within host-microbe relationships.

Bacteria living in association with seaweeds were less dependent on type II and IV secretion systems, a potentially surprising result given that both secretion systems are important tools for transferring bacterial products to the host. The type II secretion system is common in *Pseudomonadota* and is used for the secretion of various enzymes, including carbohydrate-degrading enzymes (113). The type II secretion system is also important in the matrix proteins of biofilms (114), though it was not enriched in seaweed-associated bacteria. The type IV secretion system can directly move proteins or DNA from the bacteria into another cell (115). The role of these transport systems in free-living bacteria remains understudied (116, 117), but the genes related to type IV pili, which are known to contribute to the adherence of bacterial cells to a host (118), were increased in seaweed-associated bacteria (Fig. 4). Our findings suggest that seaweed-associated bacteria might be more dependent on other transport systems, such as type III or VI, or the *Bacteroidota*-specific type IX secretion system to exchange metabolites with their host or for biofilm formation (119).

### The limits to metabolic differentiation and complementarity

Our study did not detect significant differences in metabolic complementarity between seaweed-associated and free-living microbes when we examined the *Ectocarpus* genome. If there was co-adaptation between the host and the associated bacteria that favored metabolic complementarity, the signal was too weak to be detected with this data set. Our bacterial genomes from cultivable strains could also be a biased set of those that do not require the host to survive or a set of those that are readily isolated. Metabolic complementarity might also be underestimated if gene expression is responsive to host-bacterial interactions. Notably, the *Gammaproteobacteria* associated with *Ectocarpus* showed the highest metabolic complementarity and represent a class of bacteria that are frequently found in association with brown seaweed (120, 121) and have limited suggestion of pathogenicity. Another expectation is that *Flavobacteriia*, with smaller streamlined genomes (Fig. 1) and efficient degradation of algal polysaccharides (93), might have increased metabolic complementarity. However, the opposite was true in our analyses, raising the possibility that *Flavobacteriia* may engage more in competitive interactions with their algal host, rather than cooperation, as proposed by Zelezniak et al. (122) for communities with high metabolic resource overlap.

Of the 5,914 KEGG orthologs quantified across 144 bacterial genomes, there was a strong metabolic overlap between host-associated and free-living bacteria, with some notable exceptions discussed above. An important consideration is that many seaweed-associated bacteria may exist as free-living bacteria, given that some of our algal hosts are annual species in the ocean (e.g., *Nereocystis luetkeana*) that exist seasonally as microscopic gametophytes composed of only a few cells. Although vertical transmission of bacteria in algal hosts is suggested (123), if horizontal transmission is also frequent, then bacterial genome streamlining (e.g., reference 70) should not be selected, and the maintenance of diverse metabolisms would be favored, even if costly.

In summary, we revealed strong and significant differences in the genome features and inferred metabolic capabilities of marine bacteria that were driven by evolutionary history. There were clear differences in the metabolic capabilities of seaweed-associated versus free-living bacteria. Some functions, including vitamin production and the use of seaweed-derived substrates as carbon sources, have previously been reported in algal-bacterial systems. In contrast, the differences in amino acid metabolism suggest the existence of yet-to-be-discovered interaction pathways between seaweeds and their associated bacteria, with important implications for nitrogen and sulfur cycles. Yet, the overlap we document in metabolism among free-living and host-associated bacteria suggests that bacteria associated with seaweed may not be in a close, obligate association. Alternatively, bacteria may opportunistically take advantage of predictable metabolic “leakiness” from seaweeds. Host specialization may not be selected, and bacterial generalists may experience high fitness in these marine environments (124). Our analyses necessarily contained only a subset of the likely diversity of bacteria that are associated with seaweeds, including those that may be most readily isolated, and we encourage more analyses as genomic resources emerge. The role of bacteria in seaweed host health deserves attention as coastal environments continue to experience multiple components of global change, including warming, increased variability in precipitation, and alterations to nutrients with demonstrated changes to host-associated bacteria (125, 126). As the genomes of seaweed hosts become increasingly available, we anticipate an increased understanding of reciprocal selective pressure and the traits underlying these species interactions.

## SUPPLEMENTAL MATERIAL

Supplemental material msphere.00996-24-s0001.pdf Supplemental figures and captions for supplemental tables.

Supplemental Tables msphere.00996-24-s0002.xlsx Tables S1 to S8.

ASM does not own the copyrights to Supplemental Material that may be linked to, or accessed through, an article. The authors have granted ASM a non-exclusive, world-wide license to publish the Supplemental Material files. Please contact the corresponding author directly for reuse.
