# Supplementary material for: Evolutionary history and association with seaweeds shape the genomes and metabolisms of marine bacteria
Source: mSphere. 2025 Jun 2;10(6):e00996-24. doi: 10.1128/msphere.00996-24 (PMC12188728; doi:10.1128/msphere.00996-24)
Supplement: Supplemental material — Supplemental figures and captions for supplemental tables. [file msphere.00996-24-s0001.pdf]

Supplementary Material

Supplementary Figures

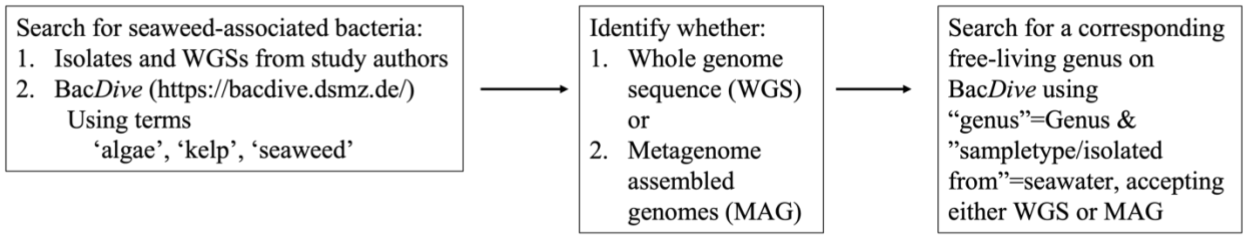

Figure S1. The workflow for identifying the genomes analyzed in the study, including all seaweed-associated or free-living. The list of all genomes and their properties is in Table S1.

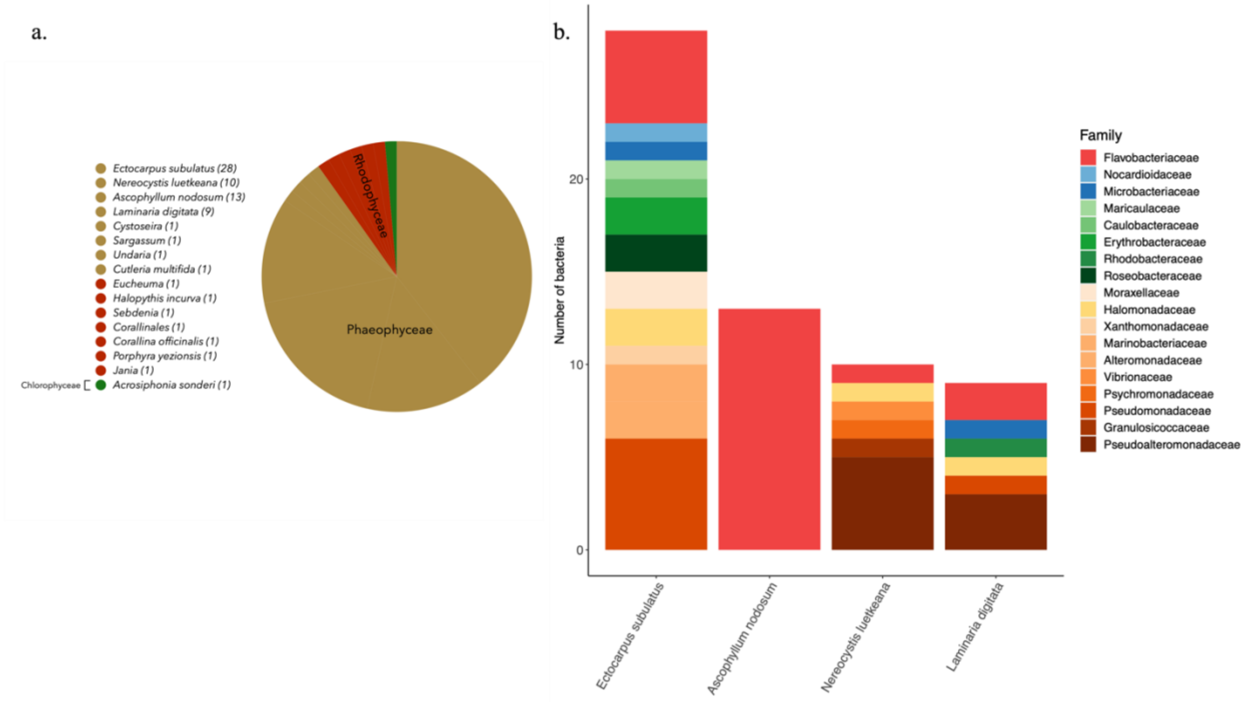

Figure S2. The distribution of hosts (A) and cultured and sequenced bacteria across the four algal species that hosted the most bacterial genomes (B) analyzed in this study.

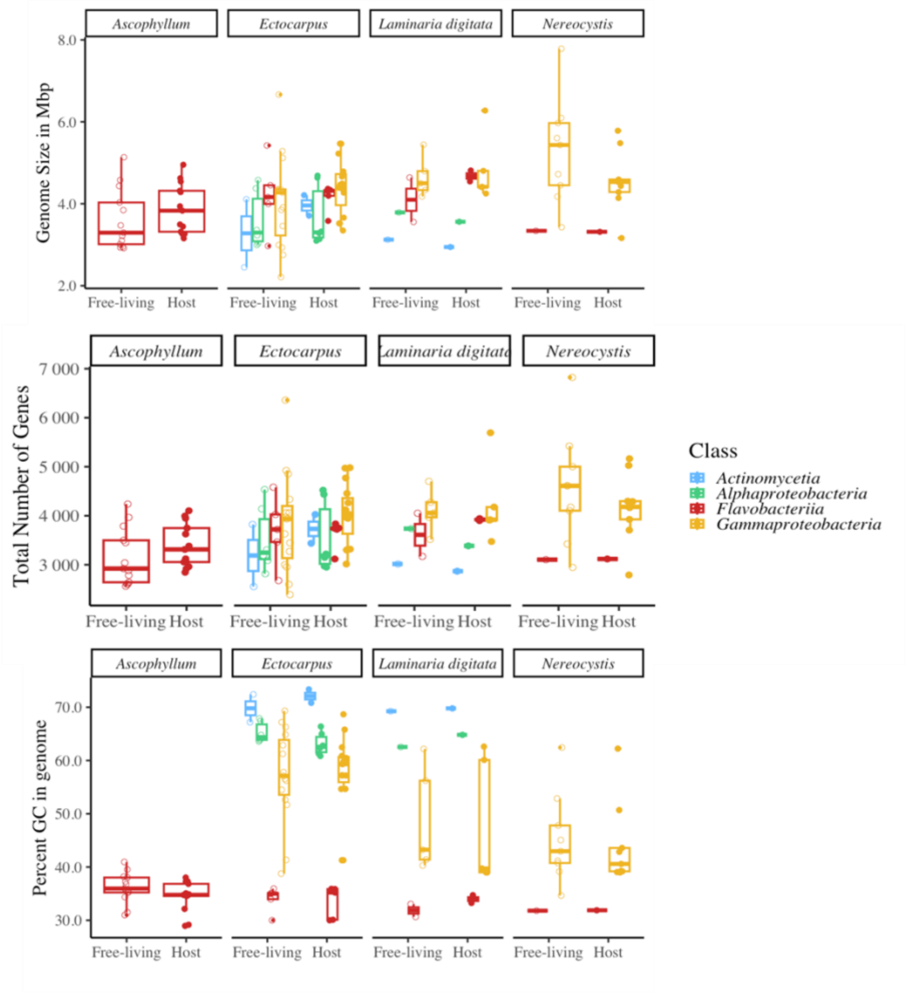

Figure S3. Features of the genomes across seaweed-associated or free-living bacteria (from Figure 1) separated by four brown algal hosts. Host seaweed had different bacterial taxa available for analysis. The genome size was lower in *Nereocystis*-associated bacteria, and GC content was lower in *Ascophyllum*-associated bacteria; otherwise, there were no differences in the genome features across algal hosts (Statistical analyses in Table S2).

Supplementary Tables (submitted in separate Excel file)

Table S1. The whole genome sequences or MAGs used in this study, including all nomenclature, their origin, and genome information. All 144 genomes are arranged by genus pairs that were either host-associated or free-living.

Table S2. Statistical analyses of the effect of bacterial taxa that were seaweed-associated or free-living on the genome size, the number of genes, and the GC content for each of the 4 brown algae hosts that had multiple associated bacteria: *Ascophyllum nodosum, Ectocarpus subulatus, Laminaria digitata,* and *Nereocystis luetkeana*. Data are shown in Figure S3. Statistical analyses for all hosts combined are shown in Tables 2 through 6.

Table S3. The statistical analyses of bacterial metabolisms by class and using PCA to test whether the bacteria that were host-associated versus free-living showed metabolic distinctness. All tests used Adonis and 999 permutations based on Bray-Curtis distances.

Table S4. The completeness of metabolic modules across all bacterial taxa estimated with 'anvi-estimate-metabolism'.

Table S5. A comparison of metabolic modules between seaweed-associated and free-living and bacteria, estimated with 'anvi-compute-metabolic-enrichment' and using the data in Table S4.

Table S6. KEGG Orthologs that were either enriched in seaweed-associated or in free-living (seawater) based on KO odds ratios in either greater than 95% or less than 5% of the distribution

all paired KO contrasts.

Table S7. KEGG Ortholog (KO) numbers that represent the 1% of genes were either enriched in seaweed-associated or in free-living bacteria in class Flavobacteriia.

Table S8. Results of a test of metabolic complementarity across all bacterial taxa associated with the brown alga *Ectocarpus subulatus.* There were 466 added-value compounds identified across 56 bacterial genomes (28 pairs).
